# How intermittent presentation affects conscious perceptual reversals of ambiguous figures

**DOI:** 10.1186/2193-1801-2-180

**Published:** 2013-04-23

**Authors:** Meihong Zheng, Kazuhiko Ukai

**Affiliations:** Department of Psychology, Tsinghua University, Haidian District, Beijing, 100084 China

**Keywords:** Ambiguous figures, Conscious perceptual reversals, netARI, D-value

## Abstract

Continually observing an ambiguous figure, we can perceive reversals between different interpretations. How perceptual reversals change when an ambiguous stimulus is presented intermittently? Since no reversal can be consciously perceived during *off-periods*, we use net Average Reversal Interval (netARI) but not usual average reversal interval to measure the perceptual reversal rate. NetARI is calculated by dividing accumulated time of *on-periods* by the number of reversals. The results are: (1) presenting an ambiguous figure intermittently increased the perceptual reversal rate; (2) the longer the exposure of Necker cube, the slower the perceptual reversal rate was, and when *on-periods* were longer as 15 s, the perceptual reversal rate was slowed down and was almost same to that in the continuous case; (3) the length of *off-periods* (which ranged from 1 s to 5 s in the present study) did not affect the reversal rate.

## Introduction

Ambiguous figures are known for their defining feature that viewers can alternately perceive two or more visual configurations during continuous observation.

According to previous studies, the perceptual reversal rate is influenced by various factors, including figure size (Washburn et al. 1931; Goldhamer 1934; Spitz and Lipman 1962), luminance (Mull et al. 1954, 1956; Heath et al. 1963), room temperature (Heath et al. 1963), and whether the two configurations are equally perceived or not (Moreno-Bote et al. 2010).

Besides visual factors, observation time (Brown 1955) and intermittent presentation (Orbach et al. 1963a; Magnussen 1972) also influence the perceptual reversal rate. Orbach et al. ( 1963b) proposed a kind of satiation mechanism to interpret the reversal phenomenon of the Necker cube. Simply put, while viewing the figure the processes mediating the perception of one orientation become satiated, and when this satiation threshold is reached a reversal is perceived. To verify their theory, they presented the Necker cube tachistoscopically using two different frequencies, and recorded the reversal rate. The reversal rate was maximal when the figure was presented at a rate of 105 exposures/min, but was reduced to near zero at 42 exposures/min. Magnussen ( 1972) also investigated the relationship between rates of intermittent presentation and perceptual reversal rates. Using a continuous presentation, and intermittent frequencies of 1, 2, 3, 5, 10 and 41 Hz, they found that reversal rates for all intermittent presentations were higher than those found using a continuous presentation mode. Furthermore, at a rate of about 2Hz the reversal rate reached its maximum value.

Contemporary research has also approached the question of how intermittent presentation of ambiguous figures affects their reversal rate with somewhat different results. Leopold et al. ( 2002) presented ambiguous figures intermittently, but at slow, non-flickering frequencies. Using this intermittent presentation method, Leopold et al. concluded that reversal s can be slowed, and even brought to a standstill. Thus, the findings of Leopold et al. contrast sharply with the enhancement of the reversal rate found with intermittent presentation of ambiguous figures in the studies performed by Orbach et al. ( 1963a) and Magnussen ( 1972).

What is the real effect of intermittent presentation on reversals of ambiguous figures? In order to clarify it, we carried out an experiment to investigate if reversals in intermittent condition were faster than that in continuous condition. Furthermore, we carried out two experiments to investigate how *off-periods* and *on-periods* influence the reversals respectively. The results help us to know more about the effects of temporal factors.

## General methods

### Measure how frequently perceptual reversals occur in intermittent conditions

The Average Reversal Interval (ARI), defined as time interval between reversals in the perception of an ambiguous stimulus, is typically used to evaluate how fast perceptual reversals occur, and calculated by dividing trail duration by the number of reversals. The calculation is undoubtedly right when a stimulus is presented continuously. However, when a stimulus is presented intermittently, it is questionable to use whole trial duration to calculate the ARI since there are exactly no reversals could be *consciously* perceived during off-periods. Take account of this, we eliminated off-periods and only use the accumulated time of on-periods to calculate ARI. In order to differentiate from original ARI, we define it as netARI. Since there is no off-period in continuous conditions, then netARIs are same to ARI in continuous conditions. In order to avoid any confusion, we call ARI as netARI even in continuous conditions.

### Analysis methods

Since different individuals have different baseline of netARI either in continuous condition or in intermittent conditions, for example, some subjects’ averaged netARIs in continuous condition are longer than 3 s, but some subjects’ ones are only less than 1 s, and in intermittent conditions, the longer ones may be 5 times of the shorter ones. In order to eliminate this baseline effect, we defined D-value as subtracting the mean of netARI differences from every netARI differences, and calculated D-value in various conditions.

### Stimuli and procedure

We used the Necker cube to be the stimulus, and it was presented on a liquid crystal monitor. The lines of the Necker cube were black and the background was white with a luminance of 125 cd/m^2^. The visual angle subtended by the cube was 4.0 degrees horizontally and 4.6 degrees vertically.

It has been observed that the perceptual reversals of ambiguous figures are initially unstable (Brown 1955). Therefore, before beginning each trial, we instructed subjects to watch the ambiguous figure for about 2 minutes, a suitable time for the perceptual reversals to be stabilized. Because the presentation of a fixation point has been shown to suppress perceptual reversals (Einhäuser et al. 2004), we instructed subjects to keep their preferred viewpoint throughout the trials, rather using an explicit fixation point. To accurately record intervals of perceptual reversals and the experiencing configuration, each participant chose two keys which represent two configurations respectively. When subjects perceive a different configuration comes to their mind, they press the corresponding key to record the configuration presence timing, then we can calculate how long a specific configuration is maintained and how often perceptual reversals occur based on intervals of two successive key presses.

In order to avoid the influence of unstable reversals in initial stage of observing a stimulus, each participant could start a trail if he or she felt reversals became stable after at least 1 minute viewing. Participants started a trail by pressing one of the two selected keys (participants were requested to select two keys in a pretrial). The duration of each trial was 60 s for both continuous condition and intermittent conditions, and each session included four trials. After each session, subjects were given a 2–3 minute break.

### Subjects

Four subjects, who could perceive reversals of Necker cube and range in age from 20 to 36 years old, participated in the experiments. Subjects provided voluntarily written consent after a full explanation of the experimental methods and procedures, but remained naïve to the purposes of the experiment and were free to withdraw at any time. Each subject had normal vision or corrected-to-normal vision.

## Experimental 1: the effect of intermittent presentation

### Methods

Aiming to know the effect of intermittent presentation, we recorded the reversal numbers in both intermittent conditions and continuous conditions. The on-periods and the off-periods changes simultaneously with a constant ratio of 2:3.

### Results

**Figure 1:**
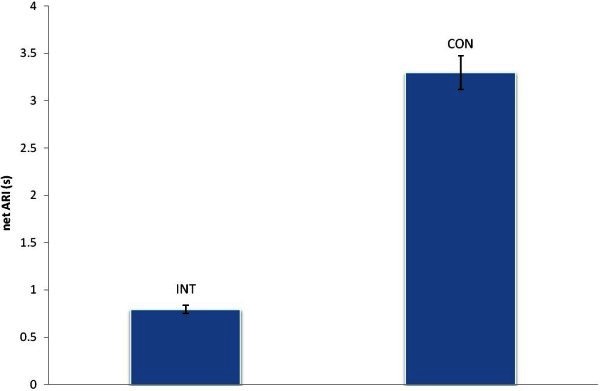
**Net Average Reversal Intervals for continuous condition and an intermittent condition in which on-period is 0.4 s, off-period is 0.6 s.** For continuous condition, Mean = 3.29, SD = 0.18, and for the intermittent condition, Mean = 0.79, SD = 0.04.

**Figure 2:**
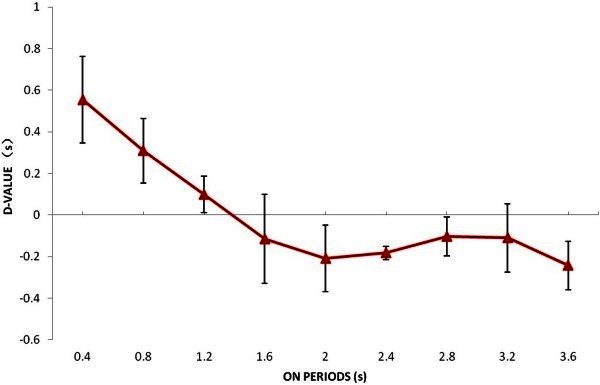
**D-value varies with on-periods when the ratio of on-period and off-period was 2:3, error bars are standard deviations.**

Figure 1 shows netARI differences between in continuous presentation and intermittent presentation for a subject. From the figure we know that the netARIs in the continuous presentation were longer than that in the intermittent presentation. The difference is statistical significant (p = 2.5E - 05) according to the result of *t*-test analysis. Figure 2 illustrates D-values in several intermittent conditions in which on-periods and off-periods vary simultaneously but keep as a constant ratio of 4:6. According to single factor ANOVA Analysis, D-values between all conditions are statistical different (F (8, 27) = 13.33916, P < 0.0001). As described in the section of analysis method, instead of netARI difference, we calculated D-values, and know from the figure that when on-periods and off-periods get bigger, D-values become smaller. Because the mean of netARI differences is 1.14 for all subjects, we know that netARIs in intermittent conditions are shorter than that in continuous conditions.

## Experimental 2: the effect of off-periods

### Methods

Since clarifying the effect of off-periods may help us to understand the role of memory in perceptual reversals, we investigated how netARIs change with off-periods first. Fixing on-period as 1 s, the number of perceptual reversals was recorded when off-periods took 1, 2, 3, 4 and 5 s, respectively.

### Results

**Figure 3:**
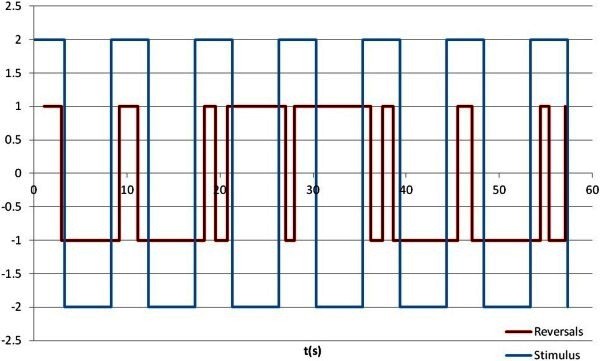
**A sample of time series of intermittent presentation and the timing of perceptual reversals.**

Stimulus presentation timing and perceptual reversals timing of a subject is shown in Figure 3. The blue square wave represents an intermittent stimulus, and the maximum represents the figure is shown, oppositely, the minimum means the figure is not shown. For observing perceptual reversals timings clearly, we chose a square which has a lower amplitude to represent perceptual configurations, one configuration is represented by the maximum, and the another one is represented by the minimum. Note that after an off-period, the configuration perceived before the off-period is perceived again.

**Figure 4:**
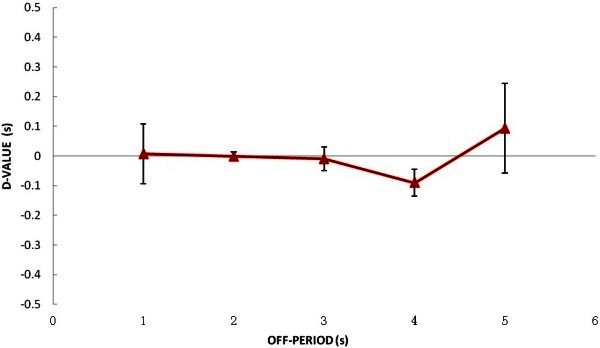
**NetARI D-values across different off-periods when on-period was 1 s, error bars are standard deviations.**

The relationship between D-value and off-periods is illustrated in Figure 4. As the figure shows, D-value curve is very flat. Furthermore, single factor ANOVA analysis reveals that there are no statistical differences between D-values for five different off-periods. Thus, we can surely conclude that netARI differences do not change with off-periods in a certain range.

Does memory only keep perception reversal process to be a continuous process, or have other roles in perceptual reversals? We will discuss the issue in the section of general discussion.

## Experimental 3: the effect of on-periods

### Methods

In this experiment, we investigated how on-periods affect perceptual reversals when off-periods were fixed. We recorded netARIs when on-periods were 1, 4, 6, 8, 10, and 15 s, and off-periods in all conditions were fixed at 5 s.

### Results

**Figure 5:**
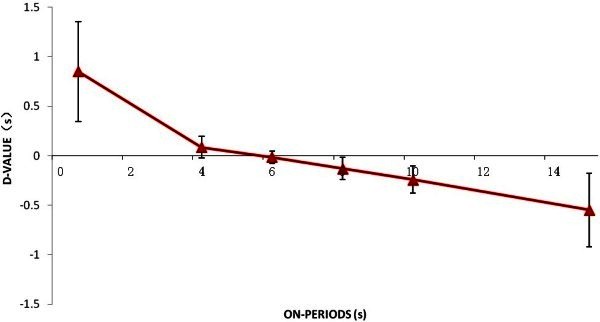
**netARI D-values across different on-periods when off-period was 5 s, error bars are standard deviations.**

The relationship between D-values and on-periods is illustrated in Figure 5. This figure illustrates that D-values get bigger with on-periods. Since the mean netARI difference of all subjects is 0.56 we know that netARIs in all intermittent conditions are shorter than that in continuous conditions. According to single factor ANOVA analysis, significant difference between netARI differences across on-periods is revealed, F (5, 18) = 12.17541, P < 0.0001. Comparing netARI differences across on-periods in intermittent presentation conditions, the longer the on-periods, the statistically longer the netARI was confirmed. Therefore, we concluded that the netARI depends only on length of on-periods and the presence of off-periods.

## General discussion

The present study clearly demonstrated several properties of perceptual reversals. First, intermittently presenting an ambiguous stimulus increases perceptual reversal rate. Second, perceptual reversal rate decreases with on-periods when an ambiguous stimulus is presented intermittently. Third, the length of the off-period (ranging from 1 s to 5 s in the present study) does not affect perceptual reversal rate.

### How intermittent presentation increases conscious perceptual reversal rate

Since only one of the two interpretations could be consciously perceived at a moment, clarifying major factors which determine how long an interpretation could be maintained is especially important. Some studies have showed that perceptual reversal rate is influenced by various factors like figure size (Washburn et al. 1931; Goldhamer 1934; Spitz and Lipman 1962), luminance (Mull et al. 1954, 1956; Heath et al. 1963), and room temperature (Heath et al. 1963). These studies provided evidence for that reversals occur during primary visual processing, but other studies supported that reversals occur during higher-level processing (for a review, see Long and Toppino 2004).

In the present study, we found that intermittently presenting an ambiguous stimulus increased the conscious perceptual reversal rate. The brain used to perceive things as meaningful things. Since an ambiguous stimulus has two or more than two different interpretations, if the brain has already recognized the all different interpretations, the different interpretations must be consciously perceived one after another, and how long one interpretation could be maintained depends on how fast the brain can or needs to reinterpret the stimulus. When an ambiguous figure is shown intermittently, the brain tries to reinterpret the stimulus within limited showing duration or under time pressure, and of course the brain reinterprets the stimulus fast. The results of experiment 3 match this analysis very well. No matter the enhancing effect of intermittent presentation caused by strengthened attention or other factors, the fact is intermittent presentation increases conscious perceptual reversal rate and the conscious reversal rate varies with on-periods of the ambiguous stimulus. About the real reasons of this enhancing effect, we will do more experiments to clarify further.

### The role of memory and the capacity of information processing in perceptual reversal process

Memory has been an issue related to perceptual reversal process. Some studies have suggested that perceptual reversals are irrelevant to memory (Borsellino et al. 1972; Fox and Herrmann 1967). Nearly one decade ago, Leopold et al. ( 2002) challenged this notion, but the role of memory in the perceptual reversal process remains unclear. In the present study, we found that memory has a role of keeping the interpretation across off-period. As Figure 2 shows, the interpretation before an off-period can be maintained even after the off-period.

Figure 5 shows the trend that netARI in intermittent presentation condition is closing to that in continuous presentation condition. This possibly because when a stimulus is shown continuously, although the input information is truly successive, but visual system divides the continuous input into several sections, so that the system can deal with limited information. The section length of continuous condition may strongly correlate with the capacity of information processing of perceptual reversals. We assume that not only for perceptual reversal process, but also for general visual perception, the system deals with information by dividing continuous input into several sections. The capacity of information processing in perceptual reversal process may relate to working memory. More experiments are needed to support this idea.
